# TikTok Influence on Rates of Tonsillectomies for Tonsil Stones

**DOI:** 10.7759/cureus.37957

**Published:** 2023-04-21

**Authors:** Nisha Bharat, Morgan Sandelski, Samantha Cerasiello, Agnes Hurtuk

**Affiliations:** 1 Otolaryngology, Loyola University Chicago Stritch School of Medicine, Maywood, USA; 2 Otolaryngology, Loyola University Medical Center, Maywood, USA; 3 Otolaryngology - Head and Neck Surgery, Loyola University Medical Center, Maywood, USA

**Keywords:** tonsilloliths, social media, tiktok, tonsillectomy, tonsil stones

## Abstract

Introduction: Tonsillectomy is one of the most common otolaryngologic surgeries and is increasingly being performed for the management of tonsil stones or tonsilloliths. Incidentally, over the years, tonsilloliths have become a popular topic on the social media platform TikTok (ByteDance, Beijing, China) and we propose that this may be influencing the trends of tonsillectomies for tonsil stones.

Objectives: We aim to assess rates of outpatient visits and tonsillectomies for tonsil stones at our institution as well as analyze videos on TikTok regarding tonsil stones.

Methods: A retrospective chart query was performed. Data including the number of patient encounters per month with a diagnosis code of tonsilloliths were collected from July 2016 to December 2021. The number of TikTok videos under the search result “tonsil stones” and the content of these videos were reviewed.

Results: There were 126 patients seeking evaluation for tonsil stones with an average age of 33.4 years, and 76% were females. The number of patients who underwent a tonsillectomy for tonsil stones increased from two in the first full year of collection in 2017 to 13 in 2021. Similarly, the average number of patients presenting for tonsil stone evaluation per month increased steadily from 1.0 in 2017 to 3.3 in 2021. TikTok video content under the search result “tonsil stones” varied and the number of videos on this topic has increased in recent years.

Conclusion: Rates of patients seeking tonsillectomy for tonsil stones increased from 2016 to 2021 in conjunction with the rising popularity of TikTok. Given the numerous TikTok videos featuring tonsil stones, we believe that this social media platform may be influencing the number of patients seeking evaluation for tonsil stones. This data may be used to understand future influence patterns of social media posts on healthcare consumer behavior and patient care practices.

## Introduction

This article was previously presented as a poster at the American Academy of Otolaryngology-Head and Neck Surgery, and Foundation (AAO-HNSF) on September 11, 2022, in Philadelphia, PA.

Tonsillectomies are one of the most frequently performed ambulatory surgeries in the United States; the most prevalent indications for tonsillectomy include recurrent tonsillitis and obstructive sleep-disordered breathing. Less commonly, tonsillectomies are performed for other indications, such as tonsilloliths [1]. Tonsilloliths stem from the accumulation of caseous debris within the crypts of the palatine tonsils. While benign, patients generally do note their presence and often complain of halitosis, cough, and occasional throat discomfort [2]. Tonsilloliths are usually managed with home treatments such as gargling or with the use of water flossers. While the COVID-19 pandemic led to a marked decrease in overall outpatient visits in all specialties including otolaryngology [3,4], a recent article noted a subjective increase in patients seeking pediatric otolaryngology evaluation for tonsilloliths during the pandemic and associated this trend with the increased usage of TikTok (ByteDance, Beijing, China) [5]. 

TikTok is a social media platform that allows users to create and view short videos on an extensive variety of topics. TikTok is currently available in approximately 150 countries and boasts over 1 billion active users worldwide [6]. In August 2018, TikTok was made available in the United States and it was noted that its popularity greatly increased during the initial months of the COVID-19 pandemic [7]. TikTok has already been used widely in the medical community by both patients and healthcare providers to share their own experiences with various medical conditions and treatments. 

When searched on the TikTok platform, the hashtag #tonsilstones has 2.6 billion views with the most prevalent videos accumulating millions of views. The content varies, but most videos include users posting videos showing their own tonsil stones, removing their tonsilloliths, and discussing extraction guides and tips. Some videos also involve medical professionals commenting further about tonsil stones. Given the growing popularity of TikTok, we sought to examine recent trends in clinic visits related to tonsil stones and subsequent rates of tonsillectomies associated with this diagnosis at our institution.

## Materials and methods

A single institution retrospective chart review was performed on otolaryngology clinic visits from July 2016 to December 2021. Electronic medical records and billing/practice management systems were queried for visits and tonsillectomies related to ICD-10 code J35.8 (other chronic diseases of tonsils and adenoids) during this time period, and these charts were reviewed for diagnosis of tonsil stones or tonsilloliths. 

Data collected included demographics, date of initial evaluation, duration of symptoms, symptomatology, medical and surgical history, smoking status, diagnostic testing, interventions prior to evaluation, physician-recommended interventions, follow-up rate, subsequent tonsillectomy, date of surgery, and timing of surgery. Descriptive statistics were used to analyze the data. 

In addition, we explored the publicly available tonsil stone and tonsillectomy-related hashtags and videos from TikTok. Content from other social media platforms was not included in this study. Some of the most popular hashtags on TikTok on this topic included #tonsils, #tonsilstones, and #tonsilstonesremoval with over 2.8 billion views. We detected 333 videos on TikTok when searching “tonsil stones”. Using this search result, we examined the 20 most-liked videos created during our study period from July 2016 to December 2021 as well as the number of videos posted per annual quarter intervals from October 2019 to March 2023. This search was conducted in March 2023.

## Results

There were a total of 126 patients with a tonsil stone diagnosis between July 2016 and December 2021; 18 patients had a mental health component documented, which included those with a diagnosis of depression, anxiety, bipolar disorder, or adjustment disorder. Additionally, 68 patients had at least one other otolaryngologic condition. The demographics of these patients are summarized in Table 1.

**Table 1:** Demographics of patients presenting for tonsil stones

Year	# of patients	Average Age (years)	% female	% male	Age at time of tonsillectomy (mean)
2016	11	23.2	91%	9%	25.5
2017	12	31.9	75%	25%	13.5
2018	18	34.6	89%	11%	37
2019	20	30.1	75%	25%	22.6
2020	25	34.4	72%	28%	27.7
2021	40	35.1	70%	30%	26.5
Overall	126	33.4	76%	24%	26.8

**Figure 1:**
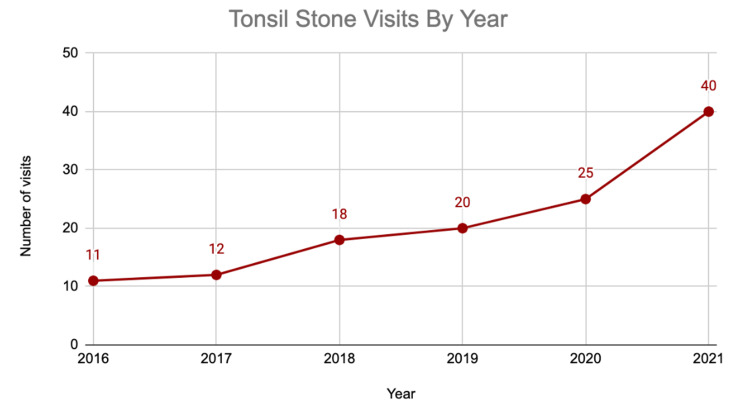
Number of outpatient visits per year with a diagnosis of tonsilloliths

**Figure 2:**
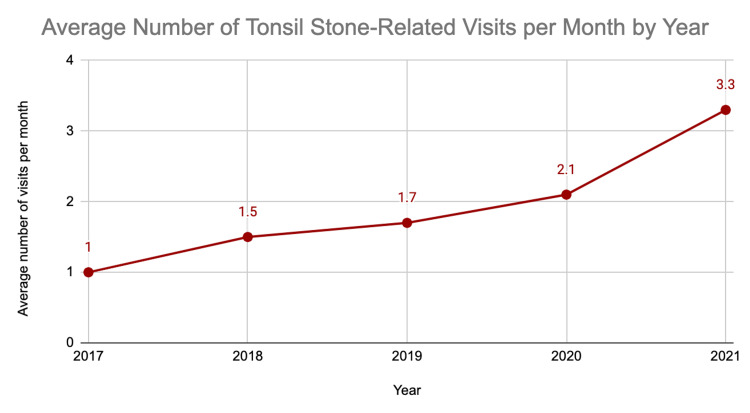
Average number of patients per month presenting for tonsil stones

The number of patients presenting with tonsil stones increased annually, from 11 patients in 2016 to 40 in 2021 (Figure 1). Similarly, the average number of patients per month seen for tonsil stones increased from 1.0 in 2017 to 3.3 in 2021 (Figure 2).

**Figure 3:**
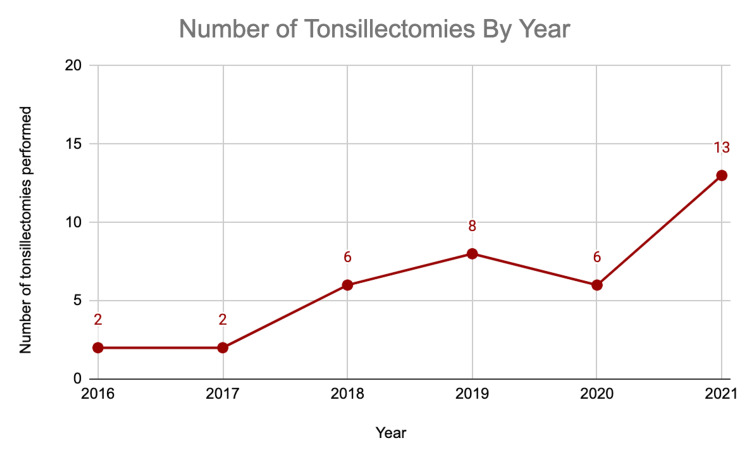
Number of tonsillectomies performed per year for the diagnosis of tonsilloliths

Of the 126 total patients seeking evaluation for tonsil stones, 37 (29.4%) patients underwent a tonsillectomy. The average age of a patient that underwent a tonsillectomy for tonsil stones was 26.8 years, and 81% of patients were females. Twenty-two (59.5%) of those patients who underwent tonsillectomies had persistent tonsil stones for at least one year at the time of their initial evaluation. The number of tonsillectomies performed for a diagnosis of tonsil stones increased yearly, from two patients in 2016 to 13 in 2021 (Figure 3).

**Figure 4:**
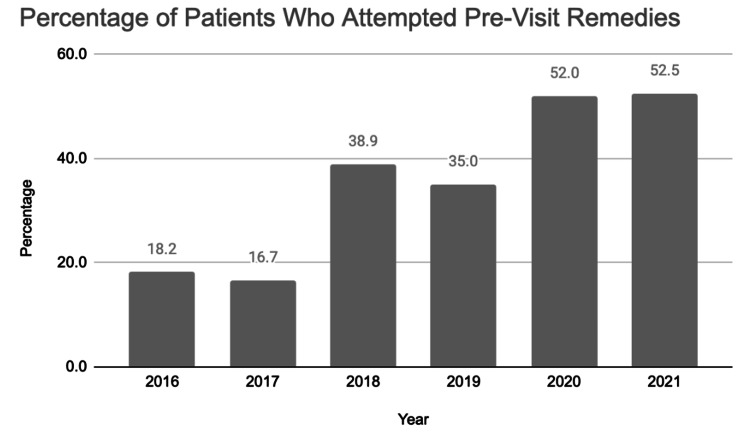
Number of patients who attempted pre-visit remedies for tonsil stones

The number of patients who attempted tonsil stone remedies prior to their clinic visit also increased from 18.2% of patients in 2016 to 52.5% in 2021 (Figure 4). The most common pre-evaluation remedies included gargling, using mouthwash, and manually picking out tonsil stones.

**Figure 5:**
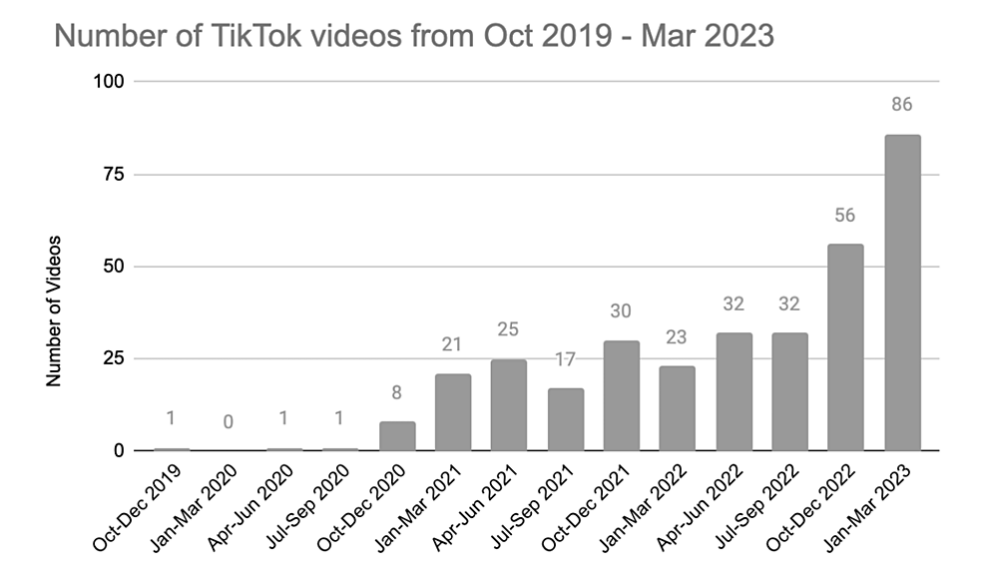
Number of videos posted per quarter on TikTok under the search result “tonsil stones”

In addition, TikTok videos on the topic of tonsil stones were analyzed. When searching “tonsil stones” in TikTok, there were a total of 333 videos available, with 104 videos created between October 2019 to December 2021 and the remaining 229 videos posted after January 2022. We analyzed the 20 most-liked videos created during our study period of 2016-2021, all of which were created in 2021. Of the top 20 videos, physician users made up 45% of video creators. Video content varied and included personal stories about tonsil stones, tonsil stone removal, and education on tonsil stones and management options (Table 2). Using the same “tonsil stones” search results, we analyzed the number of videos posted per annual quarter from October 2019 to March 2023, which showed an overall upward trend (Figure 5).

**Table 2:** Overview of the 20 most liked TikTok videos before Dec 2021 with the search result “tonsil stones”

	Number of likes (thousand)	Date posted	User Type	Video Content
1	4,100	5/11/21	Physician	Education on tonsil stones management
2	2,300	11/8/21	Nonphysician individual	Tonsil stones removal
3	1,900	5/29/21	Nonphysician individual	Tonsil stones removal
4	1,400	7/12/21	Physician	Education on tonsil stones management
5	1,200	5/17/21	Nonphysician individual	Tonsil stones removal
6	1,100	2/2/21	Physician	Education on tonsil stones management
7	440.8	2/24/21	Physician	Education on tonsil stones
8	420.3	5/22/21	Nonphysician individual	Tonsil stones removal
9	374.2	6/24/21	Nonphysician individual	Personal story
10	367.5	5/11/21	Nonphysician individual	Personal story
11	310.3	11/6/21	Nonphysician individual	Personal story
12	260.6	8/3/21	Physician	Education on tonsil stones management
13	246.4	9/22/21	Physician	Education on tonsil stones
14	233.2	1/29/21	Nonphysician individual	Tonsil stones removal
15	221.3	8/23/21	Physician	Education on tonsil stones management
16	217.8	12/14/21	Nonphysician individual	Tonsil stones removal
17	199.7	12/8/21	Nonphysician individual	Tonsil stones removal
18	193.0	6/8/21	Nonphysician individual	Tonsil stones removal
19	178.3	1/13/21	Physician	Education on tonsil stones management
20	162.0	4/15/21	Physician	Education on tonsil stones

## Discussion

Tonsillectomy is one of the most common outpatient otolaryngology surgeries performed in the United States [1], with several indications including tonsilloliths. In this study, we noticed a steady annual increase in the number of clinic visits and tonsillectomies associated with tonsil stones at our institution over a five-year period, with the largest increase in both categories from 2020 to 2021 (Figures 1-3). The drop in tonsillectomies and subtle increase in clinic visits from 2019 to 2020 (Figure 3) may in part be due to the COVID-19 pandemic restrictions and the decrease in elective clinic visits and surgeries for a portion of that year. 

During this time period, TikTok was introduced in the United States and the application has gained notable popularity since its inception in 2017. As of January 2023, the majority of TikTok users are between 10-29 years old [8], and most patients in our study also fall in this age range. Additionally, there is no strong gender preference among TikTok users, with males making up 43% of users [8]. We saw an increase in male patients diagnosed with tonsil stones from 9% in 2016 to 30% in 2021 (Table 1). The popularity of TikTok among all early adults regardless of gender and accessibility of tonsil stone videos on the platform may have led male patients to become more aware of this diagnosis and more likely to seek evaluation compared to prior public notoriety of this benign condition. 

Our examination of videos on TikTok revealed an increasing number of tonsil stones-related videos since October 2019, which has continued to rise beyond our study period (Figure 5). This trend is likely reflective of all tonsil stone videos on TikTok. Additionally, there is both physician and nonphysician engagement with the topic of tonsil stones on this social media platform, providing the public with both educational content about tonsil stones and management options as well as showing individuals discussing and removing their own tonsil stones. These videos are widespread among TikTok users, with some receiving millions of likes. Therefore, there may be a correlation between the increasing popularity of TikTok videos on tonsil stones and tonsil stone diagnosis over the past few years. 

Additionally, there was an overall increase in patients who have tried medical treatment regimens prior to their initial otolaryngology evaluation from 2016 to 2021 (Figure 4), with a higher percentage during the COVID-19 pandemic. The easily available and abundant content on tonsil stones in social media may have led to more information about remedies and management options for the condition compared to the pre-TikTok era. Given the ease of access to information, patients may be more inclined to try home treatments for their tonsil stones prior to an evaluation by a physician. The increased number of posts regarding tonsil stones may have also increased awareness of this pathology, causing more people to want to eliminate the problem or be bothered by the presence of tonsil stones when previously they may not have been aware of it. 

Social media continues to have an increasing impact on healthcare trends, with larger organizations as well as smaller medical practices creating accounts to showcase their services and achievements to the public. The posts may range from services offered, to patient testimonials, patient reviews, and other content that practices share with the goal of informing, educating, and attracting potential patients. One study investigated parents’ perspectives on Twitter about their child’s tonsillectomies, including their personal positive and negative experiences, fears, and apprehensions, as well as questions regarding recovery and financial concerns [9]. Another study found Instagram could record patients’ experience with head and neck cancers including discussion of medical appointments and treatments, managing symptoms, and cancer screening and prevention [10]. The positive impact of social media platforms was especially highlighted during the COVID-19 pandemic when social media allowed for the sharing of symptoms, learning from other hospitals across the world, and rapidly disseminating new knowledge and policies [11] during a time when face-to-face interaction was limited, and this trend may have extended to other medical conditions as well.

Previous research has also found that social media can impact patients’ healthcare decisions such as the decision to undergo various cosmetic procedures. Users who spent a significant amount of time on social media or followed plastic surgeons on social media were more likely to desire a cosmetic surgery [12,13]. Another study found that patients ranked the internet as high as medical professionals in regard to providing educational resources on laryngectomy and that the portrayal of laryngectomy on social media may potentially influence patients’ emotions, treatment preferences, or goals [14]. Social media discussions about another otolaryngologic diagnosis, ankyloglossia, have increased over the last decade, with most posts from both clinicians and parents expressing a pro-frenotomy sentiment [15,16]. Around this same time period, studies have also noted an increase in ankyloglossia diagnosis [17] and management via frenotomy, attributing this trend to the increased positive feedback about the procedure from social media [18,19]. We may be witnessing a similar phenomenon in our study in the rates of tonsil stones diagnosis and tonsillectomies. Information surrounding tonsil stones and medical and surgical treatment that is readily available on social media platforms may be influencing our patient population and their treatment choices. 

The use of social media platforms has numerous implications for patient care and education. Researchers have used Twitter posts to help create a model to predict asthma prevalence [20]. Another study found that various medical conditions including gastrointestinal (GI) disorders, chronic obstructive pulmonary disease (COPD), hypertension, diabetes, and anxiety could be predicted and identified by the language used by Facebook users [21]. Furthermore, using social media to provide social support for patients with chronic diseases can improve patient engagement and subsequent outcomes [22]. With TikTok, the numerous videos and accounts with hundreds of thousands of viewers have raised awareness of fairly common conditions including tonsillitis and tonsil stones. John et al. evaluated the content of videos regarding tonsillectomy on TikTok and concluded that videos created by physicians as well as videos about individuals’ personal experiences with tonsillectomies can contribute to a better understanding of social media trends and patient perspectives, and thus increase public education and awareness [23]. Such interventions have successfully been observed. An education program in New Zealand was created to reduce unnecessary surgery in response to the rapidly increasing demand for frenotomies [24]. Likewise, our study raises the topic of potential future implications of educating patients on the necessity for tonsillectomies for tonsil stones and ensuring safe at-home management of tonsil stones. 

While social media can contribute to increased awareness about medical diagnoses and treatments, the quality of online content is not always comprehensive, and may not be accurate. Even in highly prevalent medical topics such as diabetes, the quality and reliability of TikTok videos vary and may not fully meet health information needs among patients with diabetes [25]. In the case of tonsil stones management, using certain information from TikTok may lead to possible tonsil injury if not done safely [5]. Thus, it is important that users are cautious in obtaining medical information from social media. 

Limitations

This study has potential limitations. Users of TikTok are not representative of the entire population of people with tonsil stones, which is a very prevalent disorder. Patients who saw a primary care physician or dentist for evaluation of tonsil stones were not included in this study. As a retrospective review, we did not know if patients were TikTok users at the time of their initial evaluation for tonsil stones, although anecdotally some have mentioned during their clinic visit that they have viewed TikTok content related to tonsil stones. 

Additionally, we were unable to obtain extensive data to evaluate the content regarding tonsil stones on TikTok, including the number of views and trends in content over time, since the search results frequently change. Of note, TikTok search results show the most recent and most liked videos so we were unable to see any videos posted before October 2019. As we conducted this search in March 2023, the videos analyzed in this study were only a limited sample of all videos previously created on the topic of tonsil stones. Yet, we can infer that the increasing trend in the number of tonsil stones-related videos in recent years can be applied to years prior and is reflective of all tonsil stone videos on TikTok. 

## Conclusions

Our study illustrates the possible influence that social media, specifically TikTok, may play on patient choices and the treatment patterns of tonsil stones. TikTok content on tonsil stones is abundant, with an increase in the number of videos created on this topic in recent years. During the same time period that TikTok has gained popularity, there has been a coinciding steady increase in the number of patients presenting for tonsil stones and electing to undergo a tonsillectomy for this condition as well as patients attempting pre-visit remedies for tonsil stones at our institution. 

Videos about tonsil stones remain a popular topic on TikTok. The accessibility and notoriety of information and videos on the TikTok platform on the topic of tonsil stones may be influencing patient awareness regarding tonsil stones and their desire to seek treatment for it. Practitioners should be aware of the current social media medical content and trends that may be influencing our patients’ behaviors and decisions.
